# A Study of Classroom Behavior Recognition Incorporating Super-Resolution and Target Detection

**DOI:** 10.3390/s24175640

**Published:** 2024-08-30

**Authors:** Xiaoli Zhang, Jialei Nie, Shoulin Wei, Guifu Zhu, Wei Dai, Can Yang

**Affiliations:** 1 Key Laboratory of Computer Science, Kunming University of Science and Technology, Kunming 650500, China; zxl_km@kust.edu.cn (X.Z.); daiwei@kust.edu.cn (W.D.); 2 School of Information Engineering and Automation, Kunming University of Science and Technology, Kunming 650500, China; niejialei1127@163.com (J.N.); 17861514516@163.com (C.Y.); 3 Informationization Construction Management Center, Kunming University of Science and Technology, Kunming 650500, China; zhuguifu@kust.edu.cn

**Keywords:** super-resolution, target detection, character interaction, classroom behavior recognition

## Abstract

With the development of educational technology, machine learning and deep learning provide technical support for traditional classroom observation assessment. However, in real classroom scenarios, the technique faces challenges such as lack of clarity of raw images, complexity of datasets, multi-target detection errors, and complexity of character interactions. Based on the above problems, a student classroom behavior recognition network incorporating super-resolution and target detection is proposed. To cope with the problem of unclear original images in the classroom scenario, SRGAN (Super Resolution Generative Adversarial Network for Images) is used to improve the image resolution and thus the recognition accuracy. To address the dataset complexity and multi-targeting problems, feature extraction is optimized, and multi-scale feature recognition is enhanced by introducing AKConv and LASK attention mechanisms into the Backbone module of the YOLOv8s algorithm. To improve the character interaction complexity problem, the CBAM attention mechanism is integrated to enhance the recognition of important feature channels and spatial regions. Experiments show that it can detect six behaviors of students—raising their hands, reading, writing, playing on their cell phones, looking down, and leaning on the table—in high-definition images. And the accuracy and robustness of this network is verified. Compared with small-object detection algorithms such as Faster R-CNN, YOLOv5, and YOLOv8s, this network demonstrates good detection performance on low-resolution small objects, complex datasets with numerous targets, occlusion, and overlapping students.

## 1. Introduction

In the context of pursuing improvements in teaching quality, understanding classroom behavior and affective states has a profound impact on student achievement and teaching effectiveness. Students’ classroom behavior not only reflects their individual learning status but is also an important indicator for evaluating teachers’ teaching effectiveness. Traditionally, teachers assess student performance through manual observation, but this method is inefficient and highly subjective. The application of computer vision and deep-learning technologies provides an efficient and objective means of analyzing classroom behavior, which is crucial for enhancing teaching efficiency. In the field of deep-learning-driven student classroom behavior recognition, current research is mainly based on facial expression, body movement, and gesture estimation. However, the complexity of real classroom environments, such as low-resolution small targets, lighting variations, occlusion phenomena, and student overlap, reduces recognition accuracy. Improving the resolution of small targets and effectively extracting interaction features [1] and suppressing irrelevant features are the key ways to improve the precision and accuracy of classroom behavior recognition. Image super-resolution reconstruction [2] is an effective means to enhance the richness of details in digital images, which can reveal the subtle features of objects more accurately. In classroom scenarios, the technique can enhance the accuracy of classroom behavior recognition by increasing the resolution of small targets to capture the activity state more precisely. In addition, the application of the attention mechanism can enhance the model’s focus on key features while reducing the interference of irrelevant backgrounds, optimizing the model’s performance in complex interactions and occlusion situations. As a result, this study refers to the current deep-learning-based classroom behavior recognition methods, incorporating the super-resolution model SRGAN and the improved target detection algorithm YOLOv8s to form a cascade detection and recognition network, which achieves the detection of six behaviors: raising the hand, reading, writing, playing with a cell phone, bowing the head, and leaning on the table. In this paper, experiments were conducted to evaluate the accuracy and processing speed of the algorithmic model using the common public datasets (SCB datasets) and the self-constructed classroom video dataset of our university, and better results were obtained.

## 2. Related Work

### 2.1. Human Body Posture Estimation Recognition

Currently, various algorithms have been used to detect and identify students’ behaviors in the classroom and to correlate and analyze them with other student data. For example, Wu et al. [1] combined the PSO algorithm with the KNN algorithm to improve teaching effectiveness, resulting in the PSO-KNN joint algorithm. They also integrated it with an emotional image-processing algorithm to construct an artificial-intelligence-based classroom student behavior recognition model. Wang Zejie et al. [2] used the OpenPose human key point detection algorithm to obtain students’ key point data, input them into a convolutional neural network for learning, obtained the posture classifier, fused with local features of interacting objects extracted by YOLOv3 algorithm, and carried out recognition and analysis of students’ behaviors. Chen et al. [3] collected seven typical classroom behavior images of 300 students and pre-processed the data, extracted the key human feature information using OpenPose technology, and then trained a deep-learning model, the VGG16 network model, suitable for students’ classroom behavior recognition by migration learning ResNet50 and the Alex Net network model, and compared the accuracy of classroom behavior recognition. Fu Rong et al. [4] proposed a classroom learning behavior analysis framework using Faster R-CNN to detect the human body; OpenPose was used to extract the key points of the human skeleton, face, and fingers, and finally, a CNN-based classifier was designed for action recognition. These methods of student behavior research based on human posture estimation are usually directly extracting sparse key point locations on the human skeleton, which is more ideal for classroom behavior recognition under specific environmental experiments, but in actual complex classroom scenarios, such as character occlusion, overlapping, complex character interactions, and students’ varying proximity and distance, these objective factors will lead to poor accuracy of behavior recognition based on human posture estimation.

### 2.2. Recognition Based on Human-Object Interactions

It has been shown that in recognizing the classroom action behavior of multiple students, the clarity of the video image is an important factor affecting the recognition accuracy; recognizing the interaction activities of the target object with the surrounding objects and extracting effective student classroom behavioral features in both the channel and spatial dimensions is an important method to enhance the robustness of the model. In recent years, researchers have been working on the detection of character interaction complexity and have made some significant progress. For example, Kolesnikov et al. [5] proposed the BAR-CNN network to encode the spatial location relationship between people and objects with the help of a chain rule decomposition probabilistic network. The Visual-Spatial-Graph Network (VSGNet) proposed by Ulutan et al. [6] better characterizes spatial relationships by constructing a graph of human-object interactions. Wang et al. [7] proposed a method based on the YOLOv5s network structure to identify and analyze student classroom behavior. The method involves using convolutional layers in YOLOv5s to extract deep features and applying Squeeze-and-Excitation (SE) attention mechanisms to reduce the weight of interpersonal interaction information during recognition. Finally, the extracted features are classified using Feature Pyramid Networks (FPNs) and Path Aggregation Network (PAN) structures. Wang et al. [8] proposed the IPNet network for predicting human-object interaction points and locating and classifying interaction relationships. The above research methods mainly cut through the interaction relationship by extracting the appearance features and spatial relationship between people and objects, but they lack attention to focusing on the important features of people’s interaction, suppressing irrelevant information in the background, varying the distance of students, and enriching the degree of detail of small targets, and there is still a large potential to improve the recognition accuracy. Considering that YOLOv8s network has significant advantages in recognition accuracy, detection speed, and character interaction, this study proposes a student classroom behavior recognition model incorporating the super-resolution network SRGSN based on YOLOv8 and optimizes and improves the target detection module and the character interaction relationship construction link, so as to realize classroom behavior recognition. The main contributions of this study are as follows:

- SRGAN (Image Super Resolution Generative Adversarial Network) is used to generate the original image with high resolution, enrich the degree of detail of small targets, enhance the spatial features of the human–object interaction relationship, and improve the accuracy of recognition;
- A variable kernel convolution AKConv is added in the Backbone module of the target detection algorithm YOLOv8s, where the variable kernel convolution can adjust the initial rule pattern of adopting the network, according to the actual needs of adjusting the shape and size of the samples, so as to enable the network to adapt to different datasets and detect more targets;
- In the SPPF of the Backbone module of YOLOv8s, the integration of the LASK attention mechanism expands the receptive field and acquires wider contextual information, which significantly improves the feature aggregation capability of the SPPF module at multiple scales. It makes the network more focused on target-related features, which in turn improves the detection accuracy;
- By introducing the CBAM attention mechanism, the input features are processed through both channel and spatial dimensions, helping the model better focus on important features of character interactions and suppress irrelevant background information.

## 3. Recognition Network Model Used in This Study

### 3.1. System Architecture

**Figure 1:**
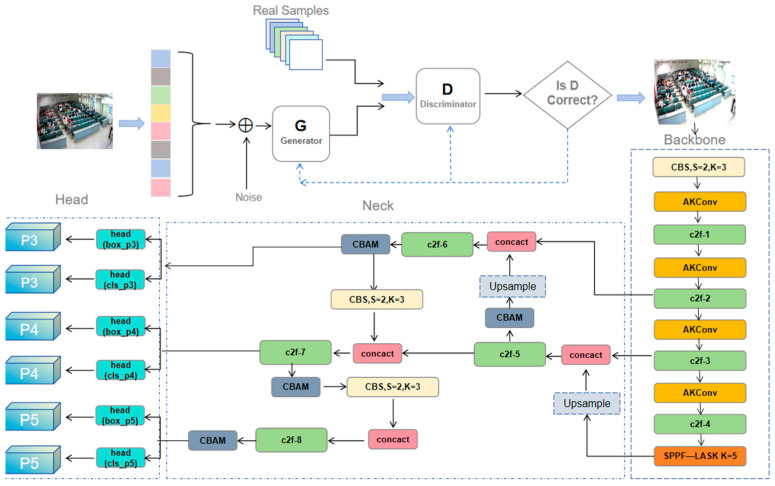
Network structure.

The network model proposed in this study is shown in Figure 1; it consists of SRGAN and the improved YOLOv8s [9] target detection network in turn. In this figure, first, SRGAN converts low-resolution classroom video frames into higher-resolution classroom video frames to generate clear classroom images. After that, the generated high-definition classroom images are used as inputs to the improved YOLOv8s by combining the variable kernel convolution and attention mechanism. Finally, six behavioral classifications of students are obtained.

The classroom behavior recognition task involves extracting character features as well as detecting and classifying targets. First, a Backbone network composed of CBS convolutional modules, the variable kernel convolution module AKConv, the C2F module, and the improved feature pyramid SPPF-LASK module in sequence is used to extract features from high-resolution images. The CBS module consists of three core components: Conv2d, BatchNorm2d, and the SiLU activation function. In the Neck network, an FPN structure incorporating the attention mechanism CBAM is used to fuse multi-scale feature maps to enhance semantic representation. Finally, the Head network processes the feature maps and decodes them through convolutional layers and fully connected layers to predict the target location and category.

### 3.2. Super-Resolution Generative Adversarial Networks

To solve the problem of lack of image clarity and small target details in real classroom scenes, SRGAN [10] is used. SRGAN is based on the SRResNet generative network and novel perceptual loss function, combining adversarial loss and content loss to optimize image resolution. Since the GAN network may introduce some artifacts or some strange human images, the first present experiment replaces the MSE loss with the perceptual loss in SRGAN. SRGAN optimizes the perceptual loss by GAN and trains the generative network G to deceive the discriminator D to produce super-resolution images with high perceptual quality. The mathematical expression is as follows:

(1) $$l^{\mathrm{SR}}=\underbrace{l_{X}^{\mathrm{SR}}}-\overbrace{10^{-3}\times l_{\mathrm{GEN}}^{\mathrm{SR}}}$$

where $l_{X}^{SR}$ is the content loss and $l_{GEN}^{SR}$ is the adversarial loss. The content loss consists of MSE and a certain ratio of the loss function inherent to the GAN network. Its expression is as follows:

(2) $$l_{X}^{\mathrm{SR}}=l_{\mathrm{MSE}}^{\mathrm{SR}}=\frac{1}{r^{2}}\times \frac{1}{W}\times \frac{1}{H}\sum_{x=1}^{\mathrm{rW}}\sum_{y=1}^{\mathrm{rH}}{\left(I_{x,y}^{\mathrm{HR}}-G_{\theta_{G}}{\left(I^{\mathrm{LR}}\right)}_{x,y}\right)}^{2}$$

The adversarial loss function is the form commonly used in GANs, and after minimization, the expression is as follows:

(3) $$l_{\mathrm{GEN}}^{\mathrm{SR}}=\sum_{n=1}^{N}-{\mathrm{logD}}_{\theta_{D}}\left(G_{\theta_{G}}\left(I^{\mathrm{LR}}\right)\right)$$

The purpose behind this is to allow the image generated by the generative network G to fool the discriminator D in the GAN, where the loss function parameters are solved for:

(4) θ^G=arg minθG1N∑n=1NlSR(GθG(InLR),InHR)

In the discriminator D, the solution parameters can then be transformed as follows:

(5) $$\min_{\theta_{G}}{\;\max}_{\theta_{D}}{\;E}_{{I^{\mathrm{HR}}}_{P_{\mathrm{train}\left(I^{\mathrm{HR}}\right)}}\left[{\mathrm{logD}}_{\theta_{D}\left(I^{\mathrm{HR}}\right)}\right]}+{\;E}_{{I^{\mathrm{LR}}}_{P_{G\left(I^{\mathrm{LR}}\right)}}\left[1-{\mathrm{logD}}_{\theta_{D}\left(I^{\mathrm{LR}}\right)}\right]}$$

For GAN networks, they address a min–max type problem. In Equation (5), it essentially keeps ‘$\theta_{G}$’ unchanged to learn and adjust ‘$\theta_{D}$’, with the goal of training a discriminator network ‘$\theta_{D}$’. The second experiment uses real samples and labels these samples so that the GAN can better distinguish between real images and generated images.

### 3.3. Improved YOLOv8s Network

#### 3.3.1. YOLOv8s Network

The YOLOv8 network [11], developed by Ultralytics (Los Angeles, CA, USA) in 2023, is an advanced SOTA model of the YOLO family, which improves on previous versions to enhance performance and adaptability. YOLOv8 offers five different-sized models, of which YOLOv8s was selected as the base network because of its overall superiority in terms of accuracy and speed. YOLOv8s is divided into Backbone, Neck, and Head.

**Figure 2:**
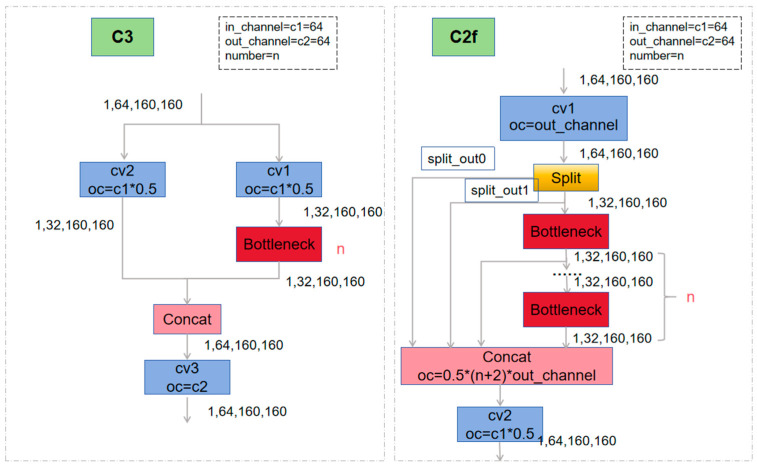
C3 and C2f network structure (Note: * denotes X).

The Backbone part, including Conv, C2f, and SPPF modules, adopts a structure similar to CSPDarknet and is responsible for extracting features from the input image. The Conv module consists of five 3 × 3 convolutional layers, which reduce the computation and obtain the global information by initial successive down-sampling. The C2f module is a customized module for YOLOv8s. Compared with the ELAN of YOLOv7 and the C3 structure of YOLOv5 as shown in Figure 2, C2f provides more hopping layer connections and channel fine-tuning to facilitate feature fusion and extraction.

**Figure 3:**
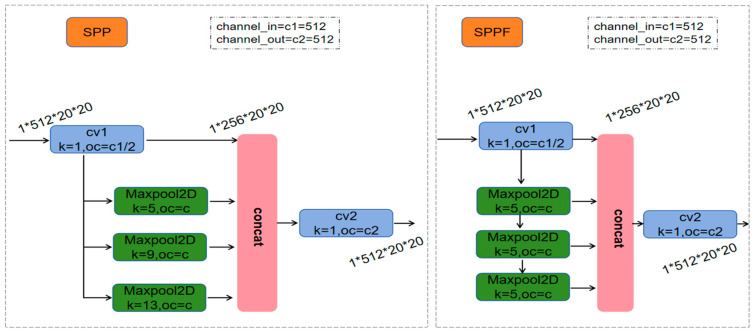
SPP and SPPF network structure.

The SPPF module, i.e., spatial pyramid pooling, is shown in Figure 3, which is used to aggregate features at multiple scales. Compared to SPP [12], SPPF changes the simple parallel max pooling to serial + parallel, which reduces the parameters and increases the speed of computation but does not change the computational results.

The Neck, as the part that performs feature fusion on the feature maps output from the Backbone network, includes two fully connected up-sampling C2f modules and two fully connected convolution C2f modules. The Neck part concatenates the extracted features from three different scale feature maps obtained by the SPPF module by up-sampling and outputs the first feature map (80 × 80) to the Head layer after two up-samplings and then outputs the remaining two feature maps (40 × 40 and 20 × 20) to the Head layer by down-sampling the feature maps through the CBS module.

The Head layer network is responsible for predicting the location and class of the target, and a decoupled head design is used to generate feature maps for Boxes and CLs, respectively. For example, the input P3 feature map (1, 256, 80, 80) is processed through the CBS module and the convolutional layer to generate (1, 64, 80, 80) feature maps for Box prediction and (1, nc, 80, 80) feature maps for CLs prediction, where nc denotes the number of categories. The Head layer ultimately produces three Box and three CLs feature maps.

#### 3.3.2. YOLOv8s Network with Variable Kernel Convolution

**Figure 4:**
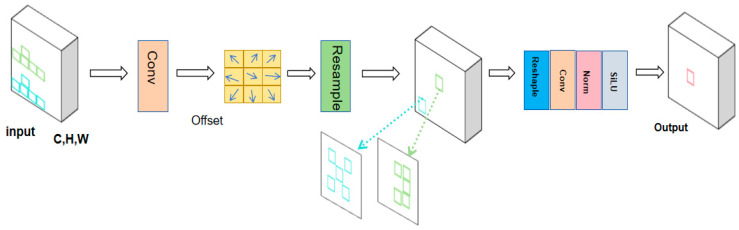
AKConv network structure.

Compared to standard convolutional operations, the variable kernel convolution AKConv [13] effectively adapts to target variations and captures information from a wider range of locations by endowing the convolution kernel with an arbitrary number of parameters and sampling shapes. The introduction of AKConv in the network as in Figure 4 aims to improve the adaptability to different datasets and target detection, thus improving the accuracy of feature extraction.

The core of AKConv lies in its initial sampling coordinate algorithm, which dynamically adjusts the sampling position of the convolution kernel to adapt to specific images and targets, thus realizing the resampling of the feature map. This process includes resampling, reshaping, re-convolution, normalization, and finally outputting the results through the SiLU activation function, aiming to improve the accuracy and efficiency of feature extraction. The results show that AKConv can effectively adapt to targets of various sizes and shapes and improve the accuracy and efficiency of feature extraction, thus enabling the convolutional neural network to extract a wider range of features and solving the problem of complex and diverse student classroom behavioral data and multiple detection targets. Therefore, this study combines the Backbone part of AKconv to YOLOv8s. In order to better fit the data and verify the validity, this study also performed auxiliary experiments by adding AKConv in different parts of the convolutional layers, respectively. Based on the experimental results (Table 2), finally, all the convolutional layers in the Backbone part of the model were replaced with AKconv, as shown in the Backbone part of Figure 1.

The initial sampling coordinate algorithm, which is based on convolutional operations to localize the features at the corresponding locations by means of a regular sampling network, is defined. The initial sampling coordinate algorithm expression is as follows:

(6) $$L_{x}=L_{0}+\Delta L_{x}$$

where $L_{x}$ is the initial coordinate for the irregular convolution, $L_{0}$ is the initial sampling coordinate, and $\Delta L_{x}$ is the adjusted coordinate change according to the offset amount. According to the weights calculated from the adjusted coordinates, the input feature maps are resampled, and finally, the resampled feature maps are subjected to a convolution operation through the convolution layer to obtain the final output. Then, the functional expression of AKConv variable kernel convolution can be defined as follows:

(7) $$\mathrm{AKConv}\left(L_{0}\right)=\sum \omega \left(L_{0}+L_{x}\right)$$

where ω denotes the convolution parameter.

#### 3.3.3. Spatial Pyramid Pooling with Attention (SPPF_LSKA)

Incorporating the LSKA [14] attention mechanism improves SPPF and reduces classroom behavior recognition errors. LSKA captures extensive contextual information through a large convolutional kernel and improves multi-scale feature extraction. YOLOv8 handles multi-scale features of SPPF more efficiently, and LSKA weights feature maps so that the network focuses on target-relevant features and improves detection accuracy.

**Figure 5:**
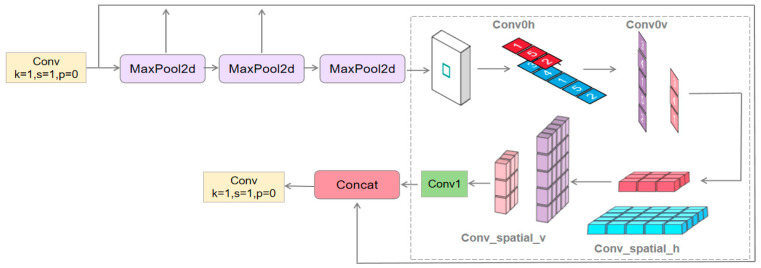
SPPF_LSKA network structure.

When adding the LSKA attention mechanism to the model, if it is added at the initial stage, although it can enhance the feature expression power, it may prematurely limit the feature learning in the subsequent layers, and it is suitable for the case of a small amount of data or a single feature. Adding it after intermediate layers such as the C2f or Conv module can balance the feature extraction and expression ability, but the features are not sufficiently multi-scale processed and may not utilize the potential of LSKA to deal with complex features. The SPPF module provides rich multi-scale features, and the addition of LSKA can enhance the detection accuracy by filtering the reinforced task-related information through the attention mechanism. The experiment compares the effect of adding LSKA in each part; the results are shown in Table 3, and the results show that the best position is shown in Figure 5 after all maximal pooling layer operations and before the fully connected layer in SPPF.

The network receives the previous layer of feature maps as input to provide the basis for the LSKA attention mechanism. The input feature map is processed by horizontal and vertical convolutional layers to extract features in horizontal and vertical directions, respectively, to generate a preliminary attention map. Then, LSKA further extracts features by spatially expanding the convolution with different expansion rates to cover a larger receptive field and capture a wider range of contextual information. These operations enhance the model’s understanding of image spatial relationships. Finally, the features are fused through the Conv1 convolutional layer to generate the final attention map. This attention map is subjected to an element-level multiplication operation with the original input feature map, so that each element in the original feature map is weighted according to the value of the attention map, and the resulting weighted feature map is passed as the output of the LSKA module to the subsequent layers of the network. These feature maps contain richer and more precise information, which helps to reduce the error in model identification.

#### 3.3.4. Improved Feature Fusion Section

The feature fusion component [15] of YOLOv8 is critical to network performance, combining different levels of feature information to account for both detailed and global information. However, in the case of complex classroom behavioral character interactions, fusing this information simultaneously increases computational complexity and may cause feature mismatch problems. To solve this problem, attention mechanisms can be introduced to selectively fuse complex classroom behavioral features.

**Figure 6:**
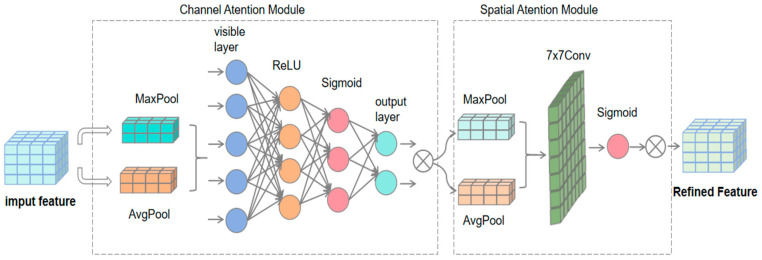
Convolution block attention module.

Attention mechanisms are usually classified into four major types: channel, spatial, temporal, and branching, but these unidirectional mechanisms cannot solve the computational and feature-matching problems caused by the complexity of classroom behavioral features. Therefore, this study introduces the CBAM [16] attention mechanism in the feature fusion part as shown in Figure 6. CBAM combines spatial and channel attention, and its lightweight design avoids extra computational burden and keeps the model efficient. In the spatial dimension, CBAM pays attention to important spatial locations and suppresses irrelevant information; in the channel dimension, it distinguishes features by calculating channel importance for better feature matching.

In this study, the CBAM [17,18,19] attention mechanism was added after the four C2f layers in the Neck section as shown in Figure 1. CBAM receives the feature maps output from the C2f [20] layer and performs global maximum and average pooling in the channel attention module to capture channel global information and lay the foundation for hierarchical feature matching. The spatial attention module also uses these two pooling operations but performs them channel by channel, and the results are concatenated and passed through the convolutional layer to generate the spatial attention weights, adjust the feature map response, and focus on the key regions. Eventually, the adjusted feature map is multiplied element by element with the original input to obtain the attention-enhanced feature map, which optimizes the channel and spatial dimensions and enriches the information for subsequent target detection tasks [21].

## 4. Experimental Results

### 4.1. Dataset

This study established an experimental dataset based on the publicly available SCB datasets and the real classroom video dataset from our university. The university’s real classroom video data were collected using a 720P camera in two classrooms, capturing six segments of 60 min each. These videos were converted into static PNG images using FFmpeg, and high-quality keyframes were selected, resulting in a total of 7378 images. The LabelImg tool was used for object annotation, defining six behavior categories: raising one’s hand, reading, writing, playing with a cell phone, looking down, and lying on the table. In total, 51,957 labels were annotated. The annotation information is saved in TXT files corresponding to the image names.

**Figure 7:**
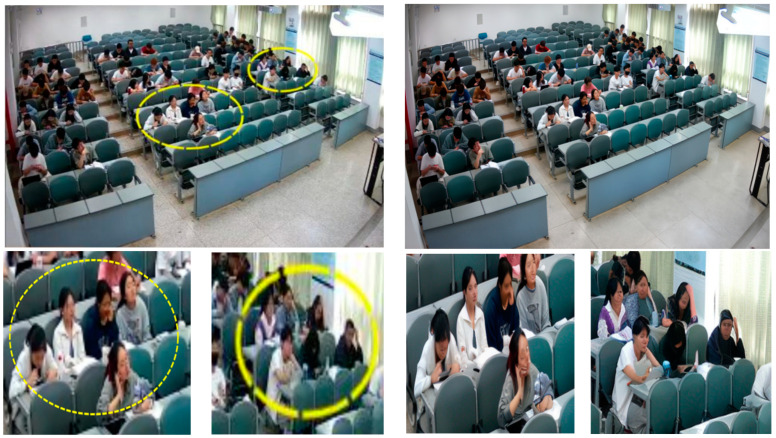
Original images and images generated by SRGAN.

After labeling, this experiment divides the labeled 51,957 datapoints into training and validation sets in the ratio of 8:2, where 41,565 datapoints are in the training set and 10,392 datapoints are in the validation set. This is quite challenging to capture the behavior in the dataset due to the complex scene environment and limited camera resolution. In order to enrich the degree of detail of small targets, enhance the spatial features of the human–object interaction relationship, and improve the model recognition accuracy, SRGAN is used to generate high-resolution images by putting the processed dataset into a one-to-one process, as shown in Figure 7, where the left column is the original image captured, and the right column is the high-resolution image generated by the SRGAN model.

Since the introduction of filmed video changes the distribution of behavioral categories and given the complexity of this network model, the model overfitting problem may be triggered in this case, and the performance may be overly optimistic especially on the training data. To suppress this overfitting, this study uses Elastic Net regularization in the classification loss function (VFL Loss), which is a combination of L1 regularization and L2 regularization. It can control the complexity and sparsity of the model and avoid the instability that L1 regularization may produce in some cases. When the dataset has a large number of features, Elastic Net can help to filter out the most important features, avoid the overfitting problem, and improve the model generalization. The mathematical formula of Elastic Net regularization is as follows:

λ1 × ∑|w| + λ2 × ∑(w^2^) (8)

where λ1 and λ2 are the regularization coefficients, and w is the model parameter. With the introduction of Elastic Net regularization, this network can effectively avoid the overfitting phenomenon when training data.

### 4.2. Experimental Environment and Configuration

The hardware platform and software version used in this experiment are shown in Table 1.

### 4.3. Indicators for Evaluation

The evaluation metrics used in this experiment include confusion matrix normalization, precision, recall, F1 score, intersection over union (IOU), mAP, mAP50, and mAP50-90 [22,23,24]. The confusion matrix provides a visual comparison of classification accuracy. Precision measures the proportion of true positives among positive cases, while recall measures the proportion of true positive examples correctly predicted. The F1 score is the harmonic mean of precision and recall. IOU measures the overlap between the predicted bounding box and the ground truth bounding box. mAP stands for mean average precision, and mAP50 and mAP50-90 denote mAP values at 50% and 50–95% IOU thresholds, respectively.

### 4.4. Experimental Results and Analysis

#### 4.4.1. Variable Kernel Convolution Based Ablation Experiments

To cope with the dataset complexity and multi-target problem, this study embeds the variable kernel convolutional AKConv into the Backbone network, feature integration layer, and detection head section of YOLOv8s and analyzes the effect of AKConv position on the evaluation metrics of the recognition task through ablation experiments. The experimental results (Table 2) show that after adding AKConv to the Backbone, SPPF, and Neck sections of YOLOv8s, all evaluation indexes are improved, proving the effectiveness of AKConv.

When all the convolutional kernels in the Backbone structure were replaced with variable kernel convolution AKConv, the evaluation metrics showed significant improvement compared to when AKConv was not added. Specifically, precision increased by 3.13%, recall increased by 2.64%, F1 value increased by 2.15%, mAP50 increased by 2.12%, and mAP50-90 increased by 3.01%. Therefore, based on these experimental results, the study incorporated AKConv into the Backbone part of YOLOv8s.

#### 4.4.2. Ablation Experiments Based on LSKA Localization

After capturing the wide range of contextual information of the image through a separable convolutional kernel, the multi-scale feature extraction capability of the model is effectively improved, and in order to maximize the utilization of these features, LSKA is added on top of the SPPF module, which further filters and strengthens the information that is helpful to the task through the attentional mechanism, thus improving the detection accuracy. In this study, positioning comparison experiments were conducted for different locations of LSKA added to the SPPF module, and the results of the experiments are shown in Table 3 below.

Bulleted

Compared to the YOLOv8s benchmark model, the detection accuracy of the classroom behavior recognition task was improved with the addition of the LSKA attention mechanism to SPPF. Experiments showed that the evaluation metrics improved most significantly when LSKA was added after all maximal pooling layers and before the fully connected layer in the SPPF module (bolded font in Table 3), so this study chose to add LSKA at this location.

#### 4.4.3. Improved Feature-Fusion-Based Partial Ablation Experiments

In the feature fusion section, features from different network layers are combined to take into account both detailed and global information. However, classroom behavioral features are complex, and simultaneous fusion may increase computational complexity and lead to performance degradation, while differences in the statistical properties of features at different layers may lead to mismatch problems. To solve these problems, an attention mechanism is introduced to selectively incorporate important features. In this study, the effectiveness of incorporating the attention mechanism was verified through comparative experiments, and the results are shown in Table 4.

lists look

As seen in the table, this study incorporates GAM [25], ECA [26], SENet [27], ShuffleAttention [28], and CBAM attention mechanisms in the feature fusion section and evaluates them by accuracy, computational resource consumption, and model complexity. The results show that the addition of the CBAM attention mechanism improves the accuracy by 4.47% over the YOLOv8s baseline without increasing the model complexity and computational resource consumption, and the GPU resource consumption is relatively small. Although the ECA attention mechanism improves the accuracy to 91.3%, its GPU resource consumption and model complexity are large, increasing the computational cost. After comprehensive comparison, this study chose to add the CBAM attention mechanism after the four C2f layers in the feature fusion section.

**Figure 8:**
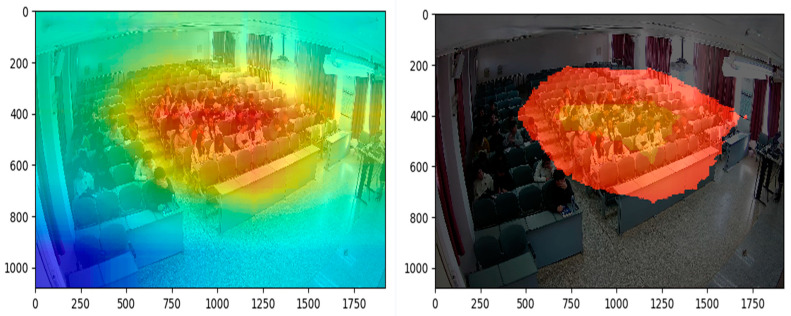
Visualization results of CBAM.

In order to further validate the role of the embedded attention mechanism CBAM, this study used the class activation graph method to visualize the attention graph, and Figure 8 shows the results of the CBAM attention visualization. In Figure 8, the original approach focuses more on the whole classroom and ignores the character interactions around the aggregated students due to the aggregation of characters and the larger background area; however, the approach focuses on where the students are aggregated through both spatial and channel dimensions, which makes the network pay more attention to the contextual cues around the students and thus improves the recognition performance.

#### 4.4.4. Comparative Experiments with Different Models

**Figure 9:**
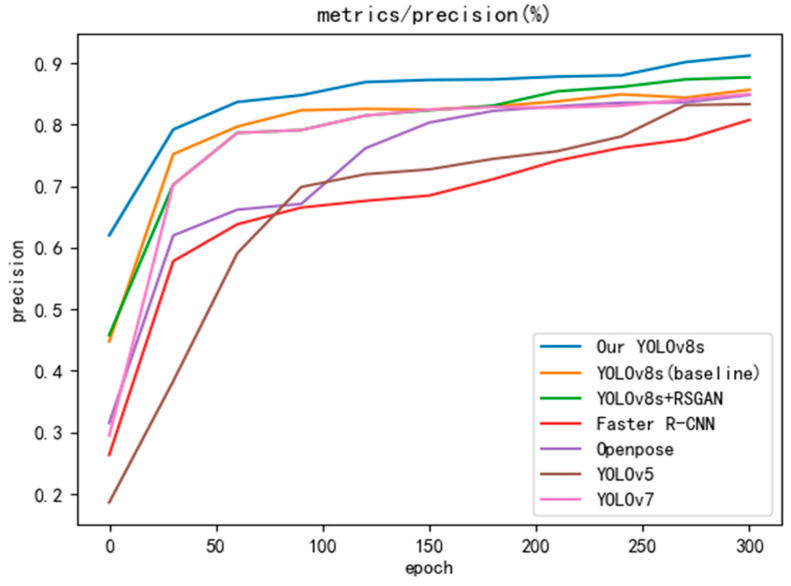
Comparative experimental results of precision.

**Figure 10:**
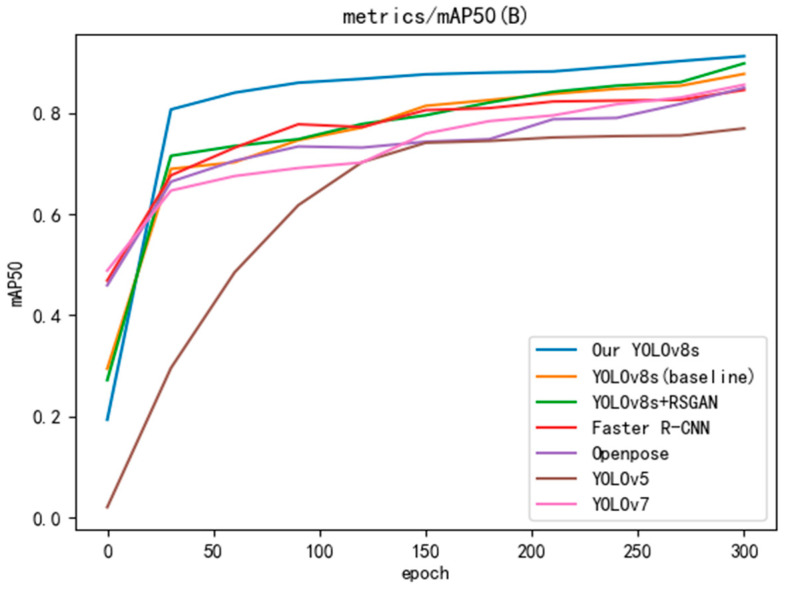
Comparative experimental results of mAP50.

To verify the superiority of the improved algorithm, YOLOv8s, Faster R-CNN [29], OpenPose [30], YOLOv5 [31], and YOLOv7 [32] are selected for comparison experiments in this study, keeping the same base parameters. The experiments were conducted using accuracy and mAP50 metrics, and values were taken at 30-round intervals to plot the curves, and the results are shown in Figure 9 and Figure 10. The improved YOLOv8s outperforms the other models in both accuracy and mAP50, and the accuracy and mAP50 are improved by 2% and 2.3%, respectively, compared with the baseline YOLOv8s model after processing by SRGAN. Combining the experimental results, the model proposed in this study has the best performance, especially with a 14.2% improvement over YOLOv5 in mAP50.

In order to evaluate in depth how the improvement of the models in this study affects the recognition effect on the six categories of behaviors, the recognition rates of the above-mentioned models for the six categories of behaviors were compared separately, and the experimental results are shown in Table 5.

like

From Table 5, it can be clearly seen that the improved method of this study has considerably increased the recognition rate of hand-raising, reading, and writing behaviors, and the average recognition rate of this study’s method in the six categories of behaviors has reached more than 90% compared to several other models. In addition, from the point of view of the recognition effect on behaviors affecting the six categories of behaviors, the method has been effective and competitive in performing the multi-target detection tasks with a certain degree of effectiveness and competitiveness.

#### 4.4.5. Results and Analysis of This Experiment

**Figure 11:**
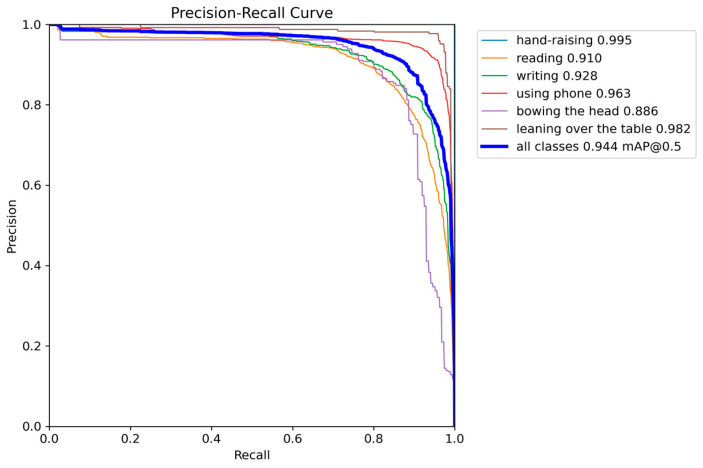
Results of the PR_Curve on the validation set.

The results of the PR_Curve (plot of precision vs. recall) for the student behavioral validation set on the proposed method in this study are presented in Figure 11.

In the PR_Curve, the horizontal axis represents recall, and the vertical axis represents precision. They often have a negative correlation; a curve closer to the top-right corner indicates that the model has high precision and high recall, meaning accurate predictions. Therefore, as shown in the figure, this research method exhibits high prediction accuracy for the validation set.

**Figure 12:**
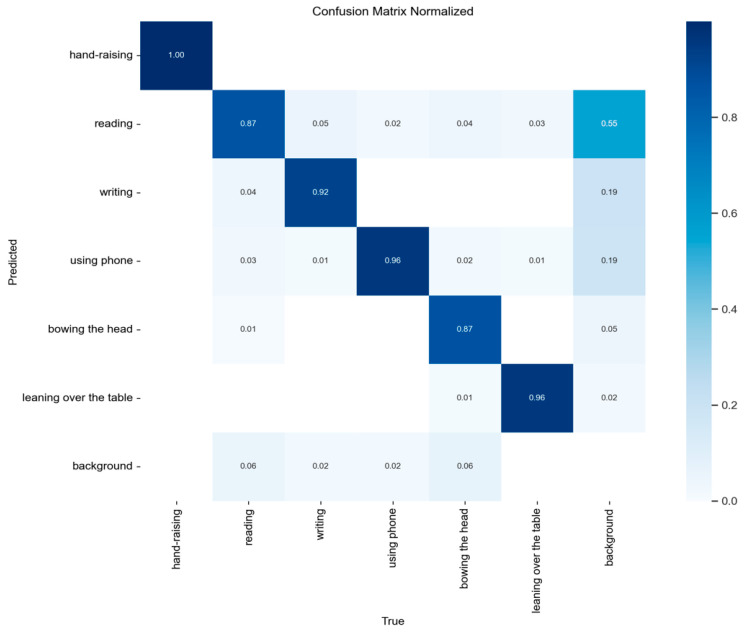
Confusion matrix with proposed method on student behavior validation.

Figure 12 shows the confusion matrix of the validation set, and the numbers on the diagonal represent the recall of each category. As can be seen from the figure, the recalls of the combinations of similar behaviors such as “reading” and “leaning over the table”, “using phone”, and “bowing the head” are higher, “bowing the head” showing the effectiveness of the proposed method in distinguishing similar behaviors. Therefore, the confusion matrix also reflects the high overall recognition rate of our method on the validation set.

**Figure 13:**
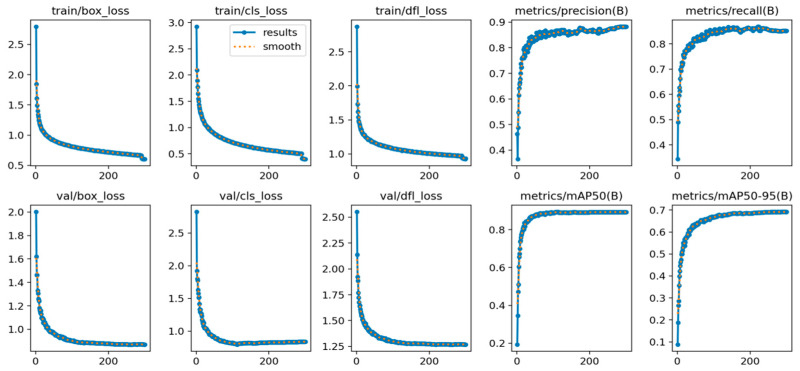
Training loss functions and performance curves.

Figure 13 shows the performance of the training and validation sets in terms of localization loss, confidence loss, and classification loss, and depicts the performance change curves for precision, recall, and average precision during training. As can be seen from the figure, the various losses decrease dramatically after a few iterations and drop to a low level and stabilize after 300 rounds of iterations. The model performance improves with the number of training rounds, stabilizes after 200 iterations, and reaches the best performance at 300 iterations. Label smoothing almost overlaps with the experimental results, indicating that the model structure is well designed without overfitting or underfitting.

**Figure 14:**
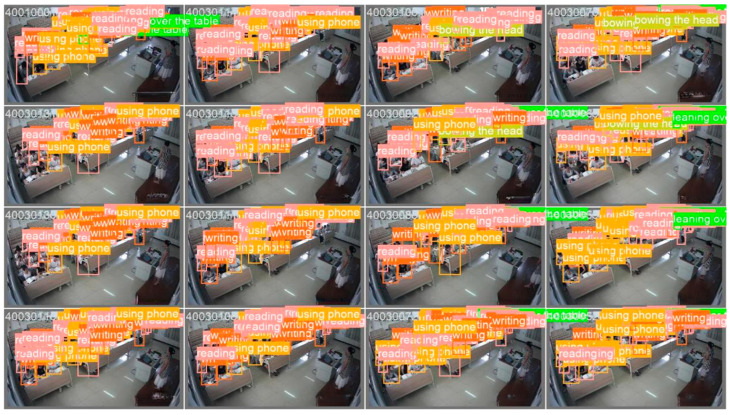
Classroom behavior detection results.

The test results of the optimized YOLOv8s model on another classroom video dataset are shown in Figure 14. Despite the challenges faced by this dataset, such as lack of clarity, target diversity, complex interactions, and object occlusion, the improved model accurately identifies student behaviors, showing excellent robustness. This improvement significantly enhances the performance of the YOLOv8s model in classroom learning behavior detection, capturing student engagement more comprehensively and accurately. This result confirms the feasibility and effectiveness of the improved YOLOv8s model in practice.

## 5. Discussion

In this paper, we use the SRGAN technique to synthesize high-resolution images as input for the recognition task. In order to improve the feature extraction capability and global and local information fusion of the YOLOv8s model, we introduce variable kernel convolution and an attention mechanism in the Backbone and Neck parts. These improvements enable the network to extract features more comprehensively and selectively fuse them according to the importance of information, effectively handling both detailed and global information. In the six student classroom behavior recognition tasks, the model demonstrates high accuracy on three behaviors: hand-raising, using one’s phone, and leaning over the table. It is also effective in recognizing reading and writing. However, due to the imbalance between behavioral categories, especially the relatively small sample size of the BOWING THE HEAD category, this can lead to significant overlap between the training and test sets. This situation tends to cause overfitting of the training data, thus affecting the generalizability of the model. To effectively address this challenge, this study introduces the Elastic Net regularization strategy, which not only reduces the risk of overfitting by imposing penalties on the model parameters but also enhances the model’s ability to recognize a small number of categories. With this regularization approach, we can control model complexity more effectively and improve its performance on unseen data. This requires future research to explore data augmentation and category balancing strategies to optimize the minority category recognition rate. The solution strategies include oversampling the minority category or undersampling the majority category, as well as assigning different weights to different categories during training to focus on the minority category and improve the model performance. It is expected that model performance will be further improved by oversampling or undersampling.

## 6. Conclusions

In response to the challenges in real classroom scenarios, this study proposes an improved YOLOv8s-based network model. The model effectively detects student classroom behaviors under conditions of insufficient image clarity, dataset diversity, and complex person interactions, demonstrating good detection results. Compared to the original YOLOv8s model, as well as Faster R-CNN, OpenPose, YOLOv5, and YOLOv7, this model shows enhanced feature extraction and recognition capabilities in complex scenarios, achieving an accuracy of 91%, with mAP50 and mAP50-95 reaching 91.5% and 70%, respectively. In the future, we plan to collect more diverse datasets to improve the model’s generalizability and applicability. Additionally, we aim to advance behavior recognition algorithms, particularly addressing issues related to small-sample learning and long-tail distribution, to enhance the accuracy of recognizing less common behavior categories.

## Figures and Tables

**Table 1:** Experimental environment.

Name	Parameter
CPU GPU	11th Gen Intel(R) Core(TM) i5-11400F @ 2.60 GHz 2.59 GHz (Intel, Santa Clara, CA, USA) NVIDIA GeForce RTX 3050 (Nvidia, Santa Clara, CA, USA)
Memory Operating System	16G Windows11
PyCharm Python	2020.1 x64 3.9.16
Frameworks CUDA	Pytorch 1.12.1 + cu113 Version: 12.0

**Table 2:** Experimental results of adding AKConv to different locations.

AKConv Position	Precision (%)	Recall (%)	F1 (%)	mAP50 (%)	mAP50-90 (%)
YOLOv8s	85.65	84.78	85.35	87.74	67.55
Backbone	88.78	87.42	87.5	89.86	70.56
SPPF	86.11	86.31	87.14	89.52	67.67
Neck	85.68	85.16	87.06	89.24	69.17
Backbone+Neck	84.45	85.21	86.12	88.12	66.78
Backbone+SPPF	85.98	84.88.	86.12	88.45	68.12

**Table 3:** Comparing LSKA positioning in the SPPF module.

LSKA Position	Precision (%)	Recall (%)	F1 (%)	mAP50 (%)	mAP50-90 (%)
YOLOv8s (baseline)	85.65	84.78	85.35	87.74	67.55
Conv_LSKA	86.25	85.21	86.35	87.98	68.12
***MaxPool2d_LSKA***	***89.46***	***88.65***	***87.43***	***90.15***	***69.97***
Concat_LSKA	87.21	86.98	87.25	88.25	68.14

**Table 4:** Experimental results of adding different attention mechanisms.

Attention	Precision (%)	GPU_Mem (G)	Params (M)	FLOPs (B)
YOLOv8s(baseline)	85.65	4.1	11.2	28.6
GAM	89.45	4.9	19.8	32.8
ECA	91.3	5.4	25.2	56.9
SENet	84.31	4.5	15.2	30.2
ShuffleAttention	88.90	4.7	17.8	34.5
**CBAM**	**90.12**	**4.6**	**17.89**	**31.54**

**Table 5:** Recognition results of student behavior using different models.

Classroom Behavior	Our YOLOv8s	Faster R-CNN	OpenPose	YOLOv5	YOLOv7
Hand-raising	**0.995**	0.842	0.836	0.884	0.841
Reading	**0.920**	0.839	0.819	0.888	0.802
Writing	**0.925**	0.814	0.789	0.892	0.782
Using phone	**0.962**	0.959	0.958	0.961	0.966
Bowing the head	**0.894**	0.901	0.888	0.842	0.905
Leaning over the table	**0.991**	0.984	0.983	0.980	0.988

## Data Availability

Data are contained within the article.
